# Understanding cervical screening non-attendance among ethnic minority women in England

**DOI:** 10.1038/bjc.2015.248

**Published:** 2015-07-14

**Authors:** L A V Marlow, J Wardle, J Waller

**Affiliations:** 1 Department of Epidemiology and Public Health, Cancer Research UK Health Behaviour Research Centre, UCL, Gower Street, London WC1E 6BT, UK

**Keywords:** pap smear, ethnicity, race, migration, attitudes, beliefs, inequalities, disparities

## Abstract

**Background:**

Women from Black, Asian and Minority Ethnic (BAME) backgrounds are less likely to attend cervical screening than White British women. This study explored sociodemographic and attitudinal correlates of cervical screening non-attendance among BAME women.

**Methods:**

Women (30–60 years) were recruited from Indian, Pakistani, Bangladeshi, Caribbean, African and White British backgrounds (*n*=720). Participants completed structured interviews.

**Results:**

BAME women were more likely to be non-attenders than white British women (44–71% *vs* 12%) and fell into two groups: the disengaged and the overdue. Migrating to the United Kingdom, speaking a language other than English and low education level were associated with being disengaged. Being overdue was associated with older age. Three attitudinal barriers were associated with being overdue for screening among BAME women: low perceived risk of cervical cancer due to sexual inactivity, belief that screening is unnecessary without symptoms and difficulty finding an appointment that fits in with other commitments.

**Conclusions:**

BAME non-attenders appear to fall into two groups, and interventions for these groups may need to be targeted and tailored accordingly. It is important to ensure that BAME women understand cancer screening is intended for asymptomatic women and those who have ceased sexual activity may still be at risk.

Cervical screening can effectively identify pre-cancerous cell changes in the cervix and these changes can be treated, preventing cancer from developing. Cervical screening is believed to have ‘prevented an epidemic' saving up to 5000 lives a year (Peto *et al*, 2004). However, the last 15 years have seen a gradual increase in the number of women who remain unscreened for 5 years or more, from 16% in 1999 to 22% in 2013 (Office of National Statistics, 1999; Health and Social Care Information Centre, 2013), and this has spurred interest in identifying the characteristics of non-attenders and their reasons for non-attendance. Population-representative surveys have suggested that non-attenders are more likely to be single and have fewer educational qualifications (Sutton and Rutherford, 2005; Marlow *et al*, 2008; Moser *et al*, 2009). In addition, women from Black and Asian Minority Ethnic (BAME) backgrounds appear to be less likely to attend screening (Sutton *et al*, 2001; Webb *et al*, 2004; Moser *et al*, 2009), and GP practices with high proportions of ethnic minority patients have lower coverage (Bang *et al*, 2012). These findings remain after adjusting for socioeconomic status (Moser *et al*, 2009; Bang *et al*, 2012). Reducing inequalities in the cancer patient pathway, including those driven by ethnicity, is a policy priority (Department of Health, 2011), and further consideration of how these inequalities can be addressed is necessary.

Ethnicity is a social construct related to race and country of birth, as well as language spoken, migration history and acculturation. The mechanisms that lead to ethnic inequalities in cancer screening uptake are likely to be complex and include both personal and organisational factors (Szczepura, 2005). Several qualitative studies have explored non-attendance at cervical screening among BAME women in England. These studies suggest a wide range of barriers including lack of knowledge about screening, low perceived risk, language difficulties, embarrassment or fear of the test, negative past experiences, negative attitudes to the NHS and practical difficulties such as time pressures (Naish *et al*, 1994; Abdullahi *et al*, 2009; Szarewski *et al*, 2009; Jackowska et al, 2012; Cadman *et al*, 2015; Marlow *et al*, 2015). While these studies have helped to identify the range of factors that might contribute to lower attendance among BAME women, quantitative work is needed to assess the prevalence of these barriers within ethnic groups and the degree to which each barrier influences uptake.

To date, few quantitative studies have explored the factors associated with non-attendance among BAME women in England. Data that are available have treated BAME women as one homogeneous non-White group (Moser *et al*, 2009) with little consideration of other ethnicity-related variables (for example, migration history and language spoken) and no assessment of attitudinal barriers. The present study aimed to explore the role of sociodemographic and attitudinal correlates in explaining cervical screening non-attendance among women from BAME backgrounds living in the England.

## Materials and Methods

We commissioned Ethnic Focus (www.ethnicfocus.com) to recruit 720 women aged 30–60 years from Indian, Pakistani, Bangladeshi, African, Caribbean and White British backgrounds (120 women from each ethnic group).

### Sampling

Data were collected using quota sampling. Sampling points (*n*=35) were randomly selected from a list compiled by Ethnic Focus of 370 post-code sectors in England, with varying concentrations of different ethnic minority groups (on the basis of census and anecdotal information). These points were inspected to ensure that they represented areas of high, medium and low concentrations of ethnic minority residents. Multilingual interviewers visited properties within each sampling point. If an eligible participant (determined by age, gender and ethnicity) lived in a household, an interview was carried out or the interviewer returned later. Three attempts were made to interview the participant before they were considered a non-responder. No incentive was offered. The study was considered exempt from needing ethical approval under the UCL Research Ethics Committee guidelines because it involved the use of completely anonymous survey and interview procedures where the participants were not defined as ‘vulnerable' and participation was not expected to induce undue stress or anxiety.

### Materials

Women completed standard closed questions with the multilingual interviewer. Questions were piloted with eight English-speaking women from BAME backgrounds before being translated into the most common languages spoken by the target ethnic groups. They were then checked for consistent meaning by bilingual researchers. Interviewers were provided with an instruction sheet clarifying any items where the meanings could be misinterpreted.

Cervical screening history was self-reported and assessed using a question based on previous work (Waller *et al*, 2009). Following the statement ‘The NHS has a cervical screening programme to prevent cervical cancer (sometimes called the smear or Pap test)' women were asked to select which of nine options best described them (multiple items could be selected): I have a smear test every year; I have had a smear test in the last 3 years; I have had a smear test but it was 3–5 years ago; My last smear test was more than 5 years ago; I have had a letter inviting me, but I did not go for the test; I have never had a letter inviting me for a smear test; I have never heard of the smear or pap test; I have smear tests in another country; I have had a hysterectomy so I don't need to have smear tests.

To assess attitudinal barriers to screening, participants were asked to respond to 11 statements using a 5-point Likert scale (strongly agree to strongly disagree); nine of these were adapted from a previous study (Waller *et al*, 2009) and two were developed from qualitative work (Marlow *et al*, 2015). Attitudinal barriers covered four broad themes; perceived need for screening, fear of cancer, concerns about the test and practical considerations.

We assessed a range of sociodemographics (age, marital status and education level) as well as migration status, language spoken and ability to read English. These items were taken from the 2011 census questionnaire. We also asked women: ‘Approximately how many times have you seen the GP (doctor) in the last 3 months' (Have not been/once/twice/3+ times).

### Analyses

Women were excluded from analyses if they reported having had a hysterectomy (*n*=11). Women who did not report attending for screening in the last 5 years were considered to be *non-attenders.* Logistic regression was used to determine the sociodemographic correlates of non-attendance, both univariable and multivariable. *Non-attenders* were then split into two groups: *overdue* (those who selected ‘My last smear test was more than 5 years ago' or ‘I have had a letter inviting me, but I did not go for the test') and *disengaged* (those who selected ‘I have never had a letter inviting me for a smear test' or ‘I have never heard of the smear or pap test'). Among the BAME sample, logistic regression was used to determine the sociodemographic correlates of being *overdue* or *disengaged* (relative to attenders). None of the white British women were disengaged.

Using *χ*^2^, we explored ethnic differences in attitudinal barriers to cervical screening, comparing each BAME group with the White British group. We excluded *disengaged* women from these analyses because we felt they may not have responded to the attitude items in a meaningful way, given that they had never heard of cervical screening/received an invitation. We tested which attitudinal barriers were associated with non-attendance, again using logistic regression.

## Results

### Sample characteristics

Overall, 1116 eligible interviewees were approached to complete 720 interviews (response rate=65%). Response rates were slightly higher for white British and Indian women (71%) than Pakistani, Bangladeshi, African and Caribbean women (61–63%). White British women were mostly married (64%) and 13% were educated to degree level. All spoke English as their first language and all were born in the United Kingdom. Most of the ethnic minority women were born outside the United Kingdom (68%); however, all women had lived in the United Kingdom for at least 5 years (mean=23 years, range: 5–54 years). Most had migrated as adults (mean age of migration: 21 years, range: 1–45 years old). Sociodemographics varied across the ethnic groups in line with population differences (see Table 1).

### Screening non-attendance

Overall, 53% of women were *non-attenders*. Women from BAME backgrounds were significantly more likely to be non-attenders than white British women (44–71% compared with 12%, see odds ratios and confidence intervals in Table 2). Older women (aged 51–60 years) were more likely to be non-attenders than younger women (30–40 years, 61% *vs* 49%). Compared with women with a degree, those who had other qualifications were more likely to be non-attenders (68% vs 56%), whereas those with some qualifications (GCSEs/A-levels/other qualification below degree level) were less likely to be non-attenders (38%). Women who had migrated to the United Kingdom as adults or children were more likely to be non-attenders than those born in the United Kingdom (64% and 65%, respectively, compared with 38%) and women who did not read English well or at all were more likely to be non-attenders (70%) than those whose main language was English (43%). In a multivariable analysis, ethnicity, age and education remained significant predictors of non-attendance.

### Sociodemographic predictors of being disengaged and overdue

**Figure 1:**
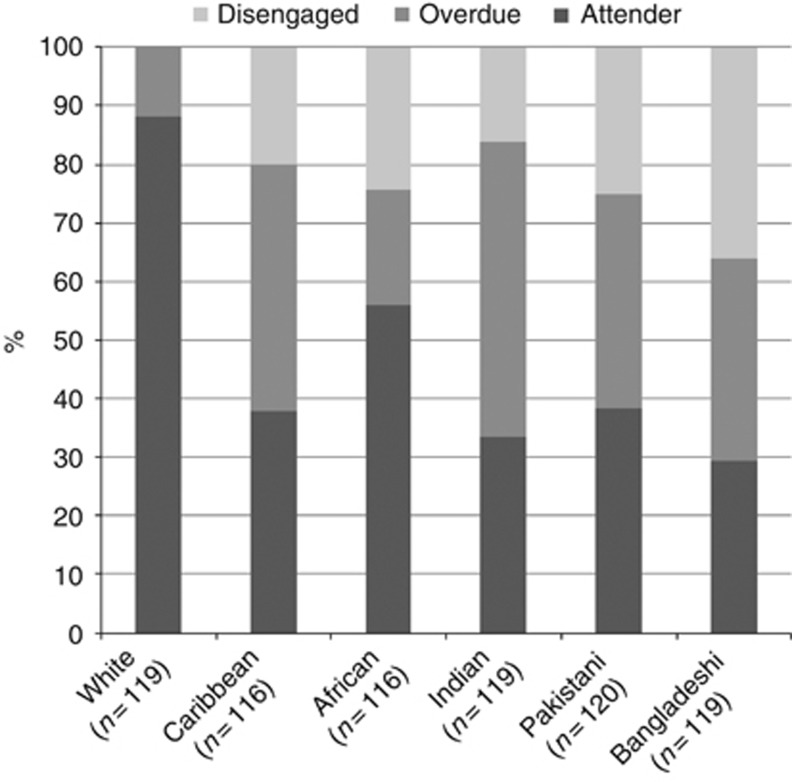
**Self-reported cervical screening status by ethnicity.**

BAME non-attenders appeared to represent two conceptually different groups (see Figure 1): *disengaged* women (those who have not heard of screening or reported never having received an invitation) and *overdue* (those who had not been screened in the last 5 years/or had not attended despite receiving an invitation). We wanted to identify which characteristics, within ethnic minority groups contributed to being *disengaged* or *overdue*. White British women were excluded from these analyses.

Overall, 24% of BAME women were disengaged. For correlates of being *disengaged*, see Table 3. BAME women who had no qualifications or ‘other' qualifications were more likely to be *disengaged* than those with a degree (31% and 37% respectively vs 13%). Women who migrated to the United Kingdom as an adult were more likely to be disengaged (33% compared with 13% of those who were born in the United Kingdom), as were those who could not read English well or at all (39% compared with 16% of those whose main language was English). In a multivariable analysis, ability to speak English was the only variable that remained significant.

Overall, 37% of BAME women were *overdue* with cervical screening. BAME women were more likely to be *overdue* if they were 51–60 years (49% compared with 32% of 30–40 year olds). No other sociodemographic variables were associated with being *overdue*.

### Attitudinal barriers to cervical screening

There were significant ethnic differences in endorsing most of the attitudinal barriers to screening (see Table 4, note, disengaged women were excluded from these analyses).

*Perceived need for screening*: Women from each ethnic minority group were more likely to think that they were not at risk of cervical cancer (25–52% *vs* 10% of white British women) and more likely to agree that they were not sexually active so did not need the test (20–42% *vs* none of the white British women). Women from Indian, Pakistani, Bangladeshi and African backgrounds were also more likely to believe that they do not need a smear test if they do not have any symptoms (57–65% *vs* 6% of white British women).

*Fear of cancer*: Compared with women from white British backgrounds, ethnic minority women were more likely to be scared of what screening might find (40–66% *vs* 24%) and more likely to say they did not want to know whether they had cancer (9–24% *vs* 3%).

*Concerns about the test:* Women from Indian, Pakistani, Bangladeshi and African backgrounds were more likely to say that they worried about seeing a male doctor/nurse (15–26% *vs* 9%) and those from South Asian backgrounds were more likely to agree that smear tests were embarrassing (71–91% *vs* 28% compared with White British women). However, BAME women were less likely to agree that smear tests are painful or that they had had a bad experience in the past, compared with White British women (pain: 13–19% *vs* 51% and bad experience: 9–14% *vs* 35%).

*Practical considerations*: Women from Indian, Pakistani, Bangladeshi and African backgrounds were more likely to say that they intend to go for screening but do not get around to it (41–48% *vs* 7% of white British women); however, there were no significant ethnic differences in endorsing family/work commitments as a barrier to screening.

We re-ran each of these analyses adjusting for (a) migration status and (b) ability to read English. All significant findings remained, suggesting that the association between ethnicity and attitudinal barriers to cervical screening is not explained by these factors. Three of the eleven attitudinal barriers were significantly associated with being *overdue* among BAME women (see Table 4): I'm not sexually active so I do not need to go for a smear test, I do not need a smear test if I do not have any symptoms and it is difficult to get an appointment that fits in with work or family commitments.

## Discussion

This is the first study to assess sociodemographic correlates of being a non-attender at cervical screening across multiple ethnic minority groups in England. It is also the first study to quantitatively compare attitudes to screening between ethnic groups. Previous studies have shown ethnic inequalities in attendance at screening, but BAME women have been considered as one homogeneous group (Sutton *et al*, 2001; Webb *et al*, 2004; Moser *et al*, 2009). We also found that BAME women were less likely to have been screened in the last 5 years than white British women and that this inequality was evident for all the BAME groups that we included. The role of ethnicity was not explained by other sociodemographic factors.

Non-attenders formed two distinct groups, ‘the disengaged' and ‘the overdue' with different sociodemographic characteristics showing associations with each group. Around a quarter of women from BAME backgrounds were ‘disengaged' from screening and these women were more likely to be from lower socioeconomic backgrounds (operationalised by education level). They were also more likely to have migrated to the United Kingdom as adults and not speak English well or at all. Around a third of the BAME women were ‘overdue' and being overdue was associated with older age. This is interesting, given that population-based studies suggest the opposite, with women from younger age groups less likely to attend screening than older women (Lancuck *et al*, 2008). Exploring the reasons for being overdue in older women is important as recent work suggests that not being screened between the ages of 50 and 64 years is associated with a greater risk of cervical cancer over 65 years (Castanon *et al*, 2014), and women over 65 years from both Asian and Black backgrounds have higher rates of cervical cancer (National Cancer Inteligence Network, 2009). Migration status and ability to read English were not associated with being ‘overdue'.

This classification of women into two distinct groups is consistent with stage theories of health behaviour (for example, the Precaution Adoption Process Model (Weinstein *et al*, 2008)). Stage theories acknowledge that there are several stages people need to move through in order to participate in a particular behaviour, with common barriers among people in the same stage, but different barriers at different stages. Classifying non-attenders into different groups has implications for how interventions might be targeted, with multiple channels likely to be the most appropriate option. For example, interventions through community groups aimed at recent migrants or English as a second language (ESOL) classes may help to identify women who are disengaged. Conversely targeting interventions towards older BAME women may help to identify those who are overdue with screening.

The content of interventions for the two groups of women may also vary. The association between language and ability to read English in the disengaged group suggests that the current written invitation is not an adequate way of engaging a significant proportion of BAME women. Women who could speak English well despite it being a second language were no more likely to be disengaged than those whose main language was English, suggesting that that ability to read screening invitations is an important factor. In addition, those migrating as children and presumably schooled at least partly in the United Kingdom were no more likely to be disengaged than those born in the United Kingdom. Many previous qualitative studies have suggested that ethnic minority groups have lower awareness of cancer screening and that poor awareness contributes to non-attendance (Abdullahi *et al*, 2009; Jackowska et al, 2012; Marlow *et al*, 2015). Our work confirms that being sent a screening letter is not sufficient to ensure that all BAME women are aware of screening and their eligibility to participate. A focus on raising awareness of screening and making it clear who is eligible may be a first step for disengaged women.

While we did not explicitly ask women whether they were registered with a GP, over 80% of the disengaged women had seen a GP at least once in the last 3 months, suggesting that they are registered to receive healthcare and should therefore have been invited for screening. Similarly, a recent study of consultation rates among cervical screening non-attenders in East London suggested many had visited a GP in the last year (Lim and Sasieni, 2015). These occasions could be opportunities for health professionals to raise the topic of cervical screening. Having said this, GP appointments with non-English-speaking patients can take longer than anticipated and likely leave little time to raise screening. An alternative strategy that does not use additional GP time but utilises the opportunity of women being at appointments could be a useful way to target the disengaged, for example, the option to see a nurse to discuss screening directly following an appointment, or provision of a video about screening in the relevant language. The possibility of offering HPV self-sampling kits to non-attenders while they are at a GP appointment has also been raised (Lim and Sasieni, 2015).

Attitudinal barriers including low perceived risk and the misperception that screening is not necessary in the absence of symptoms were associated with being overdue. This is consistent with other studies and is a cause for concern (Ackerson and Gretebeck, 2007). Information addressing the purpose of screening and the benefits of screening for women who consider themselves low risk could be beneficial. The cervical screening leaflet suggests that women who have ‘never had sex with a man or women' should ask their GP about cervical screening, but consistent messages about the relevance of cervical screening for women in long-term monogamous relationships or who have been widowed/divorced could be beneficial. Community-based interventions for non-attenders could help to increase women's understanding of the purpose of screening, particularly its aim of looking for pre-cancer in asymptomatic women. This might also help to allay fears about what screening might find, a barrier that was commonly endorsed among BAME women.

Many additional attitudinal barriers were more commonly endorsed by BAME women than White British women, consistent with findings from qualitative studies (Abdullahi *et al*, 2009; Cadman *et al*, 2015; Marlow *et al*, 2015). For example, fear of cancer was more common among BAME women and the vast majority of South Asian women found smear tests embarrassing. Although these attitudes did not seem to predict attendance (both attenders and those who were overdue found the test embarrassing and were afraid of what the results might show), minimising embarrassment and fear could help to ensure that the screening experience is acceptable to women in all ethnic groups. Reassuringly most women did not consider the screening procedure to be painful and few reported a bad experience in the past. Having other commitments was a barrier to screening for women across all ethnic groups, consistent with population-representative studies in England (Waller *et al*, 2009). Practical barriers seem to exist for all women and ways of addressing these should be considered across the board.

### Strengths and limitations

This study benefits from a large sample of women from BAME backgrounds with a range of migration histories, although it does not include recent migrants. The use of multilingual interviewers to collect the data means women who did not speak English were also included in the sample. This allowed us to consider the role of migration status and language, variables that have not received much attention in previous studies. The response rate was good, although this did vary by ethnic group and some groups (e.g., Bangladeshi) were more likely to withdraw once starting the interview. Using a questionnaire survey across a range of different ethnic groups has its limitations. In particular, cultural differences might influence the meaning participants take from a question. One example of this emerged in our original translated Bengali questionnaire where the word pray in the item ‘I would pray about a symptom before visiting my GP' was translated into ‘I would look to God', a common phase used that was not considered the same as active prayer. Although we tried to minimise discrepancies in interpretation through piloting the questionnaire with bilingual speakers and provision of notes to interviewers it became clear that there are instances where beliefs and behaviours are simply not part of all cultures, so assessing them across multiple ethnic groups may not be meaningful. In addition, it is possible that cultural norms influence social desirability particularly in face-to-face interviews with a culturally matched interviewer. Interpretation of some items such as 'I'm not sexually active so I don't need to go for a smear' should therefore be carried out with caution.

## Conclusion

BAME women who are unscreened fall into two distinct groups, and interventions for these groups may need to be both targeted and tailored accordingly. Most of the information that needs to be communicated to women is already contained in the published material that is sent alongside screening invitations, yet this does not seem to be sufficient to encourage engagement for some BAME women. Community-based interventions are likely a useful way of targeting different subgroups of BAME women. Interventions may first need to tackle awareness of screening and the availability of screening, followed by the purpose of screening and the potential benefits for women who have been in life-long monogamous relationships and are no longer sexually active. It is important to ensure that BAME women understand that cancer screening is intended for asymptomatic women and that those who have ceased sexual activity may still be at risk.

## Figures and Tables

**Table 1:** Sample characteristics by ethnic group (percentages)

	**White British (*****n*****=120)**	**Caribbean (*****n*****=120)**	**African (*****n*****=120)**	**Indian (*****n*****=120)**	**Pakistani (*****n*****=120)**	**Bangladeshi (*****n*****=120)**	***χ***^**2**^, ***P*****-value**
**Age**							
30–40	35.8	38.3	45.0	37.5	43.3	46.7	*χ*^2^(10)=9.71, *P*=0.466
41–50	35.0	35.0	38.3	39.2	35.8	35.0	
51–60	29.2	26.7	16.7	23.3	20.8	18.3	
**Marital status**							
Not married	35.8	64.2	38.3	18.3	7.5	4.2	*χ*^2^(5)=152.07, *P*<0.001
Married	64.2	35.8	61.7	81.7	92.5	95.8	
**Educational qualifications**							
No formal qualifications	0	21.7	18.3	0	24.2	20.8	*χ*^2^(15)=314.74, *P*<0.001
Some	86.7	46.7	58.9	19.2	29.2	28.3	
Degree	13.3	28.3	20.8	30.8	18.3	3.3	
Other	0	3.3	2.5	50.0	28.3	47.5	
**Migration status**							
Born in the United Kingdom	100	44.2	22.5	35.0	34.2	25.8	*χ*^2^(10)=341.85, *P*<0.001
Under 18 years	0	45.8	15.8	6.7	19.2	5.0	
Over 18 years	0	10.0	61.7	58.3	46.7	69.2	
**Ability to read English**							
Main language English	100	100	64.2	35.0	34.2	25.8	*χ*^2^(10)=347.21, *P*<0.001
Well/very well	0	0	18.3	20.0	11.7	1.7	
Not well/not at all	0	0	17.5	45.0	54.2	72.5	

**Table 2:** Non-attendance at cervical screening by sociodemographic background (*n*=709)

		**Odds ratio for non-attendance (95% confidence interval)**	
	**% Non-attenders**	**Univariable**	**Multivariable**
**Ethnicity**			
White British (*n*=119)	11.8	1.00	1.00
Caribbean (*n*=116)	62.1	12.27 (6.27–24.03)***	9.55 (4.38–20.79)***
African (*n*=116)	44.0	5.89 (3.02–11.47)***	4.73 (2.13–10.51)***
Indian (*n*=119)	66.4	14.81 (7.54–29.09)***	10.69 (4.95–23.11)***
Pakistani (*n*=120)	61.7	12.07 (6.19–23.53)***	8.09 (3.79–17.24)***
Bangladesh (*n*=119)	70.6	18.00 (9.09–35.64)***	12.86 (5.88–28.12)***
**Age**			
30–40 (*n*=294)	49.3	1.00	1.00
41–50 (*n*=259)	51.7	1.10 (0.79–1.54)	1.04 (0.70–1.53)
51–60 (*n*=156)	60.9	1.60 (1.08–2.37)*	1.95 (1.21–3.15)**
**Marital status**			
Married (*n*=511)	55.4	1.00	1.00
Not married (*n*=198)	46.0	0.69 (0.49–0.95)*	0.97 (0.64–1.48)
**Education**			
Degree (*n*=135)	56.3	1.00	1.00
Some qualifications (*n*=318)	38.7	0.49 (0.33–0.74)**	0.61 (0.38–0.98)*
No formal qualifications (*n*=99)	68.7	1.70 (0.99–2.93)	0.82 (0.41–1.62)
Other (*n*=157)	68.2	1.66 (1.03–2.68)*	0.62 (0.30–1.31)
**Migration status**			
Born in the United Kingdom (*n*=309)	37.9	1.00	1.00
Under 18 years (*n*=108)	65.7	3.15 (1.99–4.98)***	1.56 (0.83–2.94)
Over 18 years (*n*=292)	63.7	2.88 (2.07–4.01)***	1.20 (0.53–2.45)
**Ability to read English**			
Main language English (*n*=425)	42.8	1.00	1.00
Read English well/very well (*n*=59)	57.6	1.82 (1.05–3.15)*	0.96 (0.45–2.05)
Do not read English well/not at all (*n*=225)	70.2	3.15 (2.23–4.44)***	1.60 (0.74–3.46)

*P<0.05; **P<0.01; ***P<0.001.

**Table 3:** Sociodemographic predictors of being disengaged or overdue among BAME women

	**OR (95% CI)**	
	**Disengaged**	**Overdue**
**Age**		
30–40	1.00	1.00
41–50	1.07 (0.67–1.69)	1.24 (.811–1.88)
51–60	1.33 (0.74–2.41)	2.30 (1.38–3.82)**
**Marital status**		
Married	1.00	1.00
Not married	0.66 (0.41–1.07)	0.74 (0.49–1.12)
**Education**		
Degree	1.00	1.00
Some qualifications	1.09 (0.56–2.16}	0.62 (0.38–1.01)
No formal qualifications	2.94 (1.38–6.25)**	1.00 (0.54–1.85)
Other	3.41 (1.72–6.74)***	0.82 (0.47–1.43)
**Migration status**		
Born in the United Kingdom	1.00	1.00
Under 18 years	1.98 (0.99–3.96)	1.51 (0.89–2.54)
Over 18 years	3.19 (1.89–5.37)***	0.94 (0.62–1.42)
**Ability to read English**		
Main language English	1.00	1.00
Well/very well	0.66 (0.26–1.71)	1.31 (0.72–2.37)
Not well/not at all	3.58 (2.28–5.64)***	1.24 (0.82–1.88)

Abbreviations: BAME=Black, Asian and Minority Ethnic; CI=confidence interval; OR=odds ratio.

**P<0.01; ***P<0.001.

**Table 4:** Percentage agreement with attitudinal barrier statements by ethnic group

		**BAME women (*****n*****=447)**					
	**White British (*****n*****=119)**	**Caribbean (*****n*****=93)**	**African (*****n*****=88)**	**Indian (*****n*****=100)**	**Pakistani (*****n*****=90)**	**Bangladeshi (*****n*****=76)**	**OR (95% CI) for being overdue among BAME women**
**Perceived need for screening**							
I am not at risk of cervical cancer, so I don't need a smear test	10.1	24.7**	52.3***	22.0*	30.0***	30.3**	0.90 (0.61–1.35)
I'm not sexually active so I don't need to go for a smear test^a^	0.0	20.4	19.3	39.0**	37.8*	42.1**	2.60 (1.72–3.93)***
I do not need a smear test if I do not have any symptoms	5.9	5.4	56.8***	65.0***	60.0***	57.9***	1.66 (1.15–2.42)**
**Fear of cancer**							
I'm scared of what a smear test might find	24.4	45.2**	51.1***	66.0***	40.0*	43.4**	1.16 (0.80–1.69)
I don't want to know if I have cancer	2.9	8.6*	23.8***	23.8***	21.9***	19.0***	0.93 (0.60–1.44)
**Concerns about the test**							
Smear tests are embarrassing	27.7	32.3	29.5	71.0***	75.6***	90.8***	1.44 (0.99–2.11)
Smear tests are painful^a^	51.3	0	19.3***	19.0***	13.3***	17.1***	0.82 (0.48–1.41)
I've had a bad experience of a smear test in the past	34.5	14.0**	12.5**	13.0***	8.9***	10.5***	1.12 (0.63–1.98)
I am worried I will have to see a male doctor or nurse	8.9	11.5	14.6**	26.0***	20.3***	18.8***	1.00 (0.69–1.47)
**Practical considerations**							
I intend to go for a smear test but I don't get around to it	6.7	7.5	40.9***	48.0***	41.1***	46.1***	1.08 (0.73–1.58)
It is difficult to get an appointment that fits with commitments	23.6	16.2	13.1	19.4	14.7	13.1	2.20 (1.47–3.30)***

Abbreviations: BAME=Black, Asian and Minority Ethnic; CI=confidence interval; OR=odds ratio.

Analyses exclude *disengaged* women. Symbols after percentages indicate whether there was a significant difference compared with white British women.

*P<0.05; **P<0.01; ***P<0.001.

a Because no White British women agreed with this item, Caribbean women are used as the reference group.
